# Deliberating a Sustainable Welfare–Work Nexus

**DOI:** 10.1007/s11615-023-00454-6

**Published:** 2023-03-01

**Authors:** Jayeon Lee, Max Koch, Johanna Alkan-Olsson

**Affiliations:** 1 University of Gothenburg, 720, 405 30 Gothenburg, Sweden; 2 Lund University, Box 23, 221 00 Lund, Sweden; 3 Lund University, Sölvegatan 37, 223 62 Lund, Sweden

**Keywords:** Climate emergency, Safe and just operating space, Degrowth/postgrowth, Popularity of ecosocial policies, Democracy, Klimanotstand, Sicherer und gerechter Handlungsraum, Postwachstum, Popularität ökologisch-sozialer Politik, Demokratie

## Abstract

Very few countries have managed to decouple economic growth from resource use and greenhouse gas emissions in absolute terms and at rates to meet the climate targets of the Paris Agreement. To achieve this, technological solutions would need to be combined with sufficiency-oriented policies in a postgrowth context. This paper develops policy ideas for a sustainable welfare–work nexus via citizen engagement and examines the level of democratic support for such ideas. Theoretically, it employs “sustainable welfare” to understand welfare and wellbeing within planetary and social limits. The paper first sketches the welfare–work nexus as developed in the postwar circumstances in Western Europe, highlighting that this model was at no point in time ecologically generalizable to the rest of the world, and then briefly reviews the existing debate on sustainable welfare. The empirical analyses start with qualitative data from 11 deliberative forums on sustainable needs satisfaction, with emphasis on policies targeted at respecting the upper and lower boundaries of a “safe and just operating space” for economic and social development. The qualitative data are then triangulated with quantitative data from a representative survey, which was constructed based on the policy suggestions from the forums, hence allowing for an exploration of their popularity in the Swedish population as a whole. We find a considerable gap between the far-reaching policy measures that forum participants consider necessary and the measures that the general public in Sweden are prepared to support, especially when it comes to policies targeting maximum levels of needs satisfaction.

## Abstract (de)

Sehr wenigen Ländern ist es gelungen, ökonomisches Wachstum von Ressourcenverbrauch und Treibhausgasemissionen in absoluter Form zu entkoppeln oder zumindest nicht in dem Umfang, der notwendig wäre, um die Klimaziele des Pariser Vertrags einzuhalten. Soll dies dennoch zeitnah gelingen, müssten technologische mit suffizienzbasierten Ansätze in Postwachstumskontexten kombiniert werden. Der vorliegende Beitrag entwickelt und erforscht Potenziale zur Formulierung und Etablierung eines nachhaltigen Wohlfahrt-Arbeit-Nexus. Die theoretische Grundlage bildet die *nachhaltige Wohlfahrt* („sustainable welfare“) als Ansatz zum Verständnis von Wohlfahrt und Wohlempfinden innerhalb planetarer Grenzen. Wir präsentieren außerdem qualitative und quantitative empirische Daten eines gemeinsamen Forschungsprojekts in Schweden. Ausgangspunkt ist der Wohlfahrt-Arbeit-Nexus, wie er sich in Westeuropa der Nachkriegsära entwickelte und die bisherige Debatte um *nachhaltige Wohlfahrt*. Die empirischen Analysen beginnen mit qualitativen Daten von 11 deliberativen Foren zur nachhaltigen Befriedigung von Grundbedürfnissen unter besonderer Berücksichtigung von Politikvorschlägen, die auf die Einhaltung oberer und unterer Grenzen eines sicheren und gerechten Handlungsraums („safe and just operating space“) für die wirtschaftliche und soziale Entwicklung abzielen. Die qualitative Datenanalyse wird dann ergänzt durch quantitative Daten einer repräsentativen Erhebung. Der entsprechende Fragebogen wurde auf der Grundlage der Forenergebnisse erstellt, um die Popularität der dort entwickelten Politikvorschläge in der schwedischen Bevölkerung als Gesamtheit zu ermitteln. Es ergibt sich ein signifikanter Unterschied zwischen den zum Teil weitreichenden ökologisch-sozialen Maßnahmen, welche die Forumsteilnehmenden für notwendig halten, um die Klimaziele zu erreichen, und was die schwedische Bevölkerung insgesamt mitzutragen bereit ist. Dies ist besonders deutlich im Fall von Politikvorschlägen, die auf Obergrenzen bestimmter Praktiken von Bedürfnisbefriedigung abzielen.

## Introduction: Climate Emergency, Economic Growth, and Social Policy

Thresholds for biophysical processes such as the carbon and nitrogen cycles are being approached or crossed (Steffen et al. 2018), thereby influencing the potential for these processes to support human societies. The threat to global biodiversity is continuous, and the decline of biodiversity has accelerated during the last 50 years. The Intergovernmental Science-Policy Platform on Biodiversity and Ecosystem Services (IPBES 2019) highlights that decelerating this long-term trend would be a necessary precondition of maintaining human well-being. In relation to climate change, the Intergovernmental Panel on Climate Change (IPCC 2021) projects in most of its scenarios that the global surface temperature increase will exceed 1.5 °C relative to the period 1850–1900 within the next two decades. This is associated with frequent droughts, floods, and storms as well as with largely unpredictable feedback effects, considered as irreversible for centuries (Gills and Morgan 2021). In 2018, the IPCC (2018, p. 17) specified that just 12 years remained for the implementation of “far-reaching transitions in energy, land, urban and infrastructure (including transport and buildings) and industrial systems.” The task of societal transformation is becoming harder with every month without significant climate action. As global carbon emissions have since continued to increase, the window of opportunity for effective climate action has already been reduced to eight years.

In relation to achieving “net zero” economies and societies, Paterson (2021, p. 395) points out that far-reaching measures in decarbonizing electricity, electrifying ground transport, and achieving energy efficiency and technical improvements will be necessary but not suffice: Net zero means in fact “to do all of that, much more quickly, but probably eliminate ruminants (dairy as well as beef or lamb), create radical breakthroughs for production of cement, steel, and plastics, and probably to eliminate flying entirely.” The 11,000 climate researchers assembled in the Alliance of World Scientists (Ripple et al. 2020, p. 8) add in unprecedentedly clear-cut terms that, to meet the Paris targets and move toward net-zero societies, “economic growth must be quickly curtailed” to “maintain long-term sustainability of the biosphere” and that the goals of economic and other policymaking “need to shift from [gross domestic product (GDP)] growth … toward sustaining ecosystems and improving human well-being by prioritizing basic needs and reducing inequality” (Ripple et al. 2020, p. 11). This echoes recent comparative empirical studies (Haberl et al. 2020; Parrique et al. 2019) indicating that attempts to absolutely decouple GDP growth from material resource use and greenhouse gas emissions have either failed totally or did not reach the extent necessary for the large and rapid absolute reduction of carbon emissions to meet the Paris climate targets.

This paper presents results from a research project that aims at developing policy ideas for a sustainable welfare–work nexus via citizen engagement and examining the level of democratic support for them. Theoretically, it employs “sustainable welfare” (Koch and Mont 2016) as an attempt to understand welfare and well-being within planetary and social limits. Empirically, it presents qualitative and quantitative data from an ongoing research project in Sweden. The point of departure is the ecological impact of the welfare–work nexus as it developed in the postwar circumstances in Western Europe, as well as a short review of the existing debate on sustainable welfare with emphasis on moving economic and social systems toward a “safe and just operating space for humanity” (Rockström et al. 2009; Raworth 2017). The subsequent empirical analyses start with an evaluation of qualitative data from 11 deliberative forums on sustainable needs satisfaction held in 2020 in Sweden, aiming to develop key ecosocial policy suggestions arising from these forums, focusing on both the upper and lower boundaries of a safe and just operating space. This qualitative data analysis is then triangulated with quantitative data from a representative survey that included the policy suggestions from the forums, hence allowing for an exploration of their popularity in the Swedish population as a whole. The discussion and conclusion summarize the main findings and identify challenges for democratic sustainability transitions.

## From the Postwar Welfare–Work Nexus to Sustainable Welfare

After World War II, laissez-faire capitalism had lost much of its societal appeal in a range of European countries, and the entire welfare–work nexus came on the agenda. The new Fordist welfare–work nexus rested on the recognition of trade unionism and (more or less centralized) collective bargaining (Aglietta 1987). As a result, wages were indexed to productivity growth, while fiscal and credit policies were oriented toward the creation of effective demand in national economies. The trade unions, for their part, respected management’s power to control (often Taylorized) work processes. The state supported this “class compromise” by means of policies designed to integrate the circuits of the capital and consumer goods industries and by mediating conflicts between capital and labor, especially over individual and social wages. It also facilitated the provision of economic growth and productivity through public infrastructure spending and permissive credit and monetary policies. This helped production and consumption norms to increase in parallel—particularly in the case of mass-produced consumer goods of longevity such as refrigerators, televisions, cars, and standardized housing—resulting in unprecedented growth rates in GDP and real wages, particularly in the 1950s and 1960s (Koch 2017). Hence, in an attempt to combine capitalist economies and liberal democracies through the establishment of welfare systems, the state began to use the growing tax take from the primary incomes of the labour market parties to create and/or expand welfare systems to cover risks such as old age, sickness, and unemployment.

A great deal more could, of course, be said about the particular historic context in which the Fordist accumulation regime became predominant, starting with the Cold War, due to which Western wage labourers, almost by definition, had to be better off than their Eastern European colleagues (Koch 2012, pp. 49–88). The new fixation on GDP (Schmelzer 2016) played an important role in depoliticizing class conflicts and helped translate demands for democratic control of the means of production—which had been widespread after World War II, as the proposal for socialization of the energy sector and of key industries in the 1946 *Ahlen Program* of the West German Christian Democrats (the party of the chancellors Konrad Adenauer, Helmut Kohl, and Angela Merkel) illustrate—into issues of output and efficiency. Barry (2020, p. 25) retells how the West German economics minister and subsequent chancellor Ludwig Erhard (1958) was among the first to understand that instead of tiresome struggles about the distribution of a given amount of wealth, it was actually much more sensible to “grow the cake” to allow everyone a “larger slice.” And it was in the historically particular context of the *Wirtschaftswunder *(economic miracle) that the general expectation of steadily and eternally rising material living standards was created (Lutz 1989). This expansionary economic model was at the same time the structural background for the set-up of welfare institutions and social policies to provide comprehensive social security networks.

This close link between GDP growth and social welfare did not become any looser during the subsequent shift from Keynesian demand management toward “Schumpeterian” supply management (Jessop 1999) in the context of the transnationalization and financialization of production and investment. Welfare institutions were now modified and received new functions within the general structure of the “competition state” (Cerny 2010; Pedersen 2011). Designed to support competing national and/or local actors in the global economy, social policy itself came to be regarded as an investment (Hemerijck 2018). That core features of capitalist development such as unemployment ceased to be grasped as social issues and were reinterpreted as “personal problems” (Mills 1959) is expressed, for example, in the European Employment Strategy as part of the wider Lisbon Strategy. In the corresponding new social contract between individual unemployed and the state, it is the individual’s duty to look for work and to improve their adaptability to the demands of the labor market, while the state not only encourages and supports the return to the labor market of the economically inactive but also cuts or abolishes the benefits of those unwilling to do so.

Demand and supply strategies of socioeconomic regulation have in common to largely ignore the environmental aspects of welfare capitalism. Considering ecological and carbon footprints, Western material welfare standards were at no point in time generalizable to the rest of the planet (Fritz and Koch 2016; O’Neill et al. 2018)—despite the fact that these were culturally celebrated, ideologically reinforced, and exported to many other parts of the world. Indeed, had citizens of all nations led similar ways of life as Westerners, the planet would have ended up in acute climate emergency significantly earlier. Given the scarce evidence for an absolute decoupling of GDP growth, resource use, and greenhouse gas emissions, the remainder of this section addresses the welfare state’s capability of providing welfare and well-being in the absence of GDP growth and within planetary boundaries.

The framework of a safe and just operating space (Rockström et al. 2009; Raworth 2017) may serve as a point of departure since it simultaneously considers the concepts of planetary and social boundaries.^1^ Within this line of reasoning, “development” of economy and society may proceed within a doughnut-shaped space (Raworth 2017), where resource use is below planetary limits (the outer boundary or the “safe” and ecologically sustainable space) but above the sufficiency level required to meet people’s basic needs (the inner boundary or the socially “just” space). Adding to social science and political economy approaches (Brand et al. 2021; Koch and Buch-Hansen 2021; Spash 2021a) concerned with the institutional features, power asymmetries, and material interests that are inherent to an expansionist capitalist economy and that complicate a transition to the above outlined space, this framework opens up for an alternative understanding of what Spash (2021b) calls “social ecological economics.” While the latter generally focuses on the social genesis and development of an economy conceptualized as a subsystem of the planetary and social systems, welfare systems would then likewise be regarded as “embedded in the ecological context” (Hirvilammi 2020, p. 6) and grasped as “provisioning” and “appropriation systems” (Fanning et al. 2020) for sustainable need satisfiers (Max-Neef 1991). Accordingly, social policies would no longer take the relatively unproblematic form of redistributions of growing tax takes but involve, in postgrowth contexts, controversial decisions targeted at the power, resources, and interests of the rich (Corlet Walker et al. 2021; Koch 2022a).

The concept of “sustainable welfare” in general and theories of human need in particular may serve as guidelines for such a new generation of public policymaking. Sustainable welfare has been defined as meeting the needs of all people, that is, not just those of the happy few in the global North, now and in the future (Koch and Mont 2016). This systematically includes the notion that in a constrained world, not all “wants” for often “positional goods” can be politically supported in the name of consumer sovereignty. Some of these would indeed need to be restrained. Further debates about concepts of well-being and welfare within planetary and social limits have resulted in the adoption of needs-based accounts (Max-Neef 1991; Gough 2017) over hedonic, utilitarian, and subjective accounts of well-being (Büchs and Koch 2017). Max-Neef’s human scale development (HSD) methodology introduced the term “satisfier” to highlight the culturally specific ways in which universal needs are being met in practice (Guillén-Royo 2015). In Gough’s “dual strategy,” the practical knowledge of citizens complements the various sorts of expert knowledge. Using this approach, lay and expert knowledges can be brought together in participatory exercises such as workshops, consultations, or deliberative forums in order to identify alternative and sustainable needs satisfiers valid for particular communities (Guillén-Royo 2020; Temesgen 2021). From the perspective of a wider ecological and social transformation, policy ideas serving as what Max-Neef called “synergetic” needs satisfiers are particularly relevant because they have the potential of fulfilling more than just one need in different contexts and may hence serve as entry points for initiating a “virtuous policy circle of sustainable welfare” (Hirvilammi 2020).

Policy deliberations circling around the framework of a safe and just operating space and welfare provisioning systems have addressed maximum and minimum levels for needs satisfaction as well as ecosocial policy instruments with the potential of steering economy and society toward respecting such “floors and ceilings” (Gough 2020). Corresponding policy suggestions have been tabled in various areas, ranging from macroeconomic steering, inequality/redistribution via carbon rationing, and consumption to work-time regulation. There is agreement, especially among sustainable welfare scholars, that turning these policy suggestions into reality would require, on top of “bottom-up” civil society engagement (Buch-Hansen 2018; Koch 2022b), an actively intervening state (Koch 2020). Concerning the “floors” or the sufficiency level of needs satisfaction, proponents have, for example, suggested the introduction of a universal and unconditional basic income (UBI; e.g., Van Parijs and Vanderborght 2017), the expansion or introduction of universal basic services (UBS; e.g., Coote and Percy 2020), a voucher system (Bohnenberger 2020), or a combination of the three (Büchs 2021). Concerning the upper boundary, far fewer proposals have been tabled. While philosophical “limitarianism” theoretically defends the respect of limits in an ecologically constrained world (Robeyns 2019), more concrete economic proposals (Concialdi 2018; Pizzigatti 2018) suggest the definition of maximum incomes as some quantitative proportion of minimum incomes (10:1, 20:1, etc.). There is, however, no agreement about where exactly a cap (beyond which taxation would be 100%) should be set or whether all forms of wealth should be targeted (Buch-Hansen and Koch 2019).

## Deliberating Ecosocial Policies via Citizen Forums

### The Design and Implementation of Citizen Forums

Within the project “Sustainable Welfare for a New Generation of Social Policy,” we organized 11 citizen forums on sustainable needs satisfaction in 2020, with 84 participants in total. Since the main aim of these forums was to generate new policy ideas, and the generated qualitative data were subsequently triangulated with quantitative survey data (see the following section), we did not specifically aim to recruit a representative group of participants in terms of background characteristics. Instead, we engaged with individuals and groups that in a broad sense were interested in generating ideas or developing practices for sustainable welfare. We did try to include different types of social groups by, for instance, considering the number of participants residing in urban areas and in the countryside. At a later stage, we reached out to particular groups that had been relatively underrepresented in the forums that were held earlier in the research process, such as people with migrant backgrounds and younger people. Gender, family status, professions, or other socioeconomic factors were not explicitly considered in the recruitment process to the forums. However, information about occupations and residential areas was voluntarily shared by the participants during introduction rounds. While seven forums involved already established and “organized” groups of people—for example, an association organizing people without employment in Malmö—the other four forums were conducted with participants who individually registered to the forums via an open announcement. The decision to participate in our forums required rather strong commitment, as each forum lasted at least six hours (excluding breaks). We hence did not have a large pool of potential participants, and there were no selection criteria when an organization or an individual expressed their interest in participating; instead, we welcomed all individuals who volunteered. Because of the COVID-19 pandemic, only four of 11 forums took place in person, and the rest were in digital format. The Zoom meetings were recorded and transcribed by the involved researchers, who also took notes during all meetings (Table 1).

**Table 1:** List of citizen forums on sustainable needs satisfaction in Sweden (2020)

Forum no. (no. participants)	Group/individual participants	Physical/digital	Occupations represented	Place of residence
Forum 1 (6)	Community organization for people without employment	In person	Freelancer, activist, actor, journalist	Large city
Forum 2 (5)	Nongovernmental organization working with nature conservation	Zoom	Artist, teacher, activist, civil servant, retiree	Large university city, about 90,000 residents
Forum 3 (5)	Local organization—Fridays for Future	Zoom	Gardener, nurse, artist, teacher	Small city, about 7000 residents
Forum 4 (6)	Community organization working with a local grocery store	Zoom	Teacher, outdoor pedagogue, gardener, midwife, biologist	Countryside
Forum 5 (6)	Social enterprise working with migrant women	In person	Unemployed yet with former experiences as teacher, nurse, police, etc	Large city
Forum 6 (4)	Open forum in English	Zoom	Landscape architect, artist, municipal civil servant, researcher, administrator	Mostly large city
Forum 7 (13)	Open forum over two evening sessions	Zoom	Retiree, business owner, teacher, social worker, local politician, professor, philanthrope	Mostly large city
Forum 8 (5)	Open forum	Zoom	Researcher, politician, activist, entrepreneur	Mostly large city
Forum 9 (8)	Open forum in English	Zoom	Personal assistant, student, activist, teacher, business owner	Mostly large city
Forum 10 (18)	Teenagers in community education program	In person	Students aged 13–18 years	Large city
Forum 11 (8)	People engaged in local climate contract initiative in midsized city	In person	Municipal civil servant, business owner, activist, biologist, student	Midsized city, about 110,000 residents

Note: By “large city,” we mean the three largest urban areas in Sweden: Stockholm, Göteborg, and Malmö, all having more than 300,000 residents

The workshops followed Max-Neef’s Human Scale Development methodology (Max-Neef 1991), Guillén-Royo (2015). The point of departure is that all people everywhere on the planet, now and in the future, are seen as having the same fundamental needs, but these are satisfied in different ways depending on historic, social, cultural, and local contexts (Max-Neef 1991). This method aims to open up discussion for collective deliberations about alternatives, including more environmentally and socially sustainable ways of needs satisfaction.^2^ Table 2 introduces the “needs matrix” used to structure the forum discussions. First, the participants discussed the ways in which fundamental needs are currently met as well as alternative ways of meeting these needs with reduced energy use and lower ecological impacts. Max-Neef’s terms of positive, negative, and pseudo needs satisfiers were applied in this context. Second, the participants identified so-called bridging and synergetic needs satisfiers, which can facilitate the transition processes from current ways of living to a more sustainable society (Guillén-Royo 2015). We encouraged participants to focus on the policy areas of food, mobility, housing, and work life to remind participants that the forums should be grounded in everyday life. Filled-in needs matrices (Table 2) from the citizen forums and meeting notes are the main data sources of this study. The exact wording of the nine fundamental needs has been modified slightly compared to Max-Neefʼs original work because precision and additional information were needed when applying it to the Swedish context.

**Table 2:** Needs matrix by Max-Neef (1991), modified by the authors

	*Being* Physical state and mental mindset—Individual or collective	*Having* Social structures, policies, norms, and attitudes	*Doing* Individual or collective actions	*Interacting* Physical places and the social environment
*Nutrition and health*	–	–	–	–
*Protection and support*	–	–	–	–
*Proximity and love*	–	–	–	–
*Understanding and knowledge*	–	–	–	–
*Participation*	–	–	–	–
*Idleness and rest*	–	–	–	–
*Creation*	–	–	–	–
*Identity and affiliation*	–	–	–	–
*Freedom and independence*	–	–	–	–

Scholars have utilized this needs matrix in a range of ways and different contexts. These include calculations of carbon footprints relative to different satisfiers of fundamental needs (Vita et al. 2019), assessments of policy framework such as the circular economy (Clube and Tennant 2020), and identification of sustainable consumption corridors (Guillén-Royo 2020). In our citizen forums, the needs matrix was utilized as a way of structuring the deliberations, for instance by preassigning certain time slots for the discussion of specific needs. Previous applications of the fundamental needs matrix for community and/or citizen engagement focused on the establishment of consensus among participants before each cell of the matrix was filled in (Guillén-Royo 2015, 2020). However, because we ran most of the forums in digital format, we opted for a model in which the matrix was used as a way of registering all ideas that surfaced during the forums. This meant that the forum participants had more freedom in forging discussions compared to the model in which researchers aim for consensus for each cell of the matrix. The moderator’s role in the forums was limited to ensuring that all nine needs in the matrix were discussed and reminding the participants of the definitions of different types of needs satisfiers. In the later part of the forum, the moderator encouraged participants to propose “bridging” and “synergetic” needs satisfiers, which we in the analysis phase identified as policy ideas. The matrices that were filled during the forums by two researchers were added together to one single master matrix, where it was possible to filter through the identified needs satisfiers using different categories of needs as well as secondary themes developed through a thematic coding process (such as consumption, democracy, equality, work and income, and use of space).

The resulting data consist of a wide range of topics, as the method itself encourages a holistic reflection process (Guillén-Royo 2015), and the data can therefore be reorganized and presented in multiple ways. For this paper, we present policy ideas generated through the forums that are related to setting the upper, or planetary, and lower, or sufficiency, boundaries of a safe and just operating space (see previous section). In what follows, we summarize the key policy suggestions that the forum participants proposed and categorize them in relation to whether they focus on regulating maximum levels of satisfaction of needs and wants or guaranteeing minimum levels of needs satisfaction. Hence, rather than reporting our forum results using quantitative methods (for instance, by counting the number of times a given needs satisfier or policy proposal was mentioned), we present the themes of the forum discussions most salient to the main focus of this paper and present them in a brief manner.

The forum participants rarely expressed fundamentally conflicting opinions, and the discussions took place in a consensual atmosphere. The fact that we mainly worked with already established groups (Table 1) may partly explain this result. The relatively high degree of consensus could also be due to the fact that the participants were not randomly selected but participated voluntarily, indicating their individual interests in contributing to the deliberation of ecosocial policy ideas. Finally, the fact that we did not push for consensus achievement in any way, primarily using the matrix as a tool to register the results of the brainstorming of ideas, may have contributed to a generally relaxed atmosphere in which points of view were exchanged in mutual respect.

### Regulating Maximum Levels for the Satisfaction of Needs and Wants

Critically reflecting upon the ways in which fundamental needs are met today, our forum participants frequently problematized excessive consumption, regarded as unnecessary and unsustainable, and argued for heavier taxation and higher prices for airline flights in particular and for fossil fuels and energy-intensive production in general. Excessive consumption and unsustainable resource use in the richer parts of the world were seen as core problems. Related to this, it was frequently mentioned that there are still people struggling with basic needs satisfaction or sustenance in Sweden (especially in relation to the needs “nutrition and health” as well as “protection and support”), and this number is increasing. Moreover, excessive consumption was argued as hampering the satisfaction of other needs such as “participation,” “idleness and rest,” “creation,” “understanding,” and “freedom and independence.” The consumption-driven culture in general and commercials in particular were criticized for hindering people’s capacity to reflect upon genuine needs and corresponding environmentally sustainable needs satisfiers and identities less shaped by current patterns of consumption. Many participants argued that consumption was undermining the satisfaction of nonmaterialistic fundamental needs, and a less consumeristic society could leave more room for creative activities, time for rest, and energy for political and civil participation.

There was ample support for a range of ideas in relation to the identification and respect of the upper boundaries of the safe and just operating space. Several participants pointed out the problem of increasing income and asset inequalities as a detrimental factor for well-functioning democracies. To the extent that people have vastly different experiences in their everyday lives because of material inequalities, it was argued, the possibility for common political visions is becoming unattainable. The discussion about maximum level of needs satisfaction was hence not solely concerned with the consumption side. Introducing a cap on earnings was proposed, as well as an alternative remuneration system that would allow workers to take more free time as an equivalent for increased productivity at work, instead of higher salaries.

Overall, our forum participants were more prone to point out the need for guaranteeing minimum levels of needs satisfaction for all, which is described below, rather than talking about directly intervening in people’s choices regarding how they work, how much money they should earn, what they consume, etc., as this would mean disrupting many taken-for-granted norms of our current economic system head on and in radical ways. “Softer” policy ideas such as introducing advertisement-free zones in cities are telling examples where the forum participants envisioned nudging or suggested indirect mechanisms for the reduction of unsustainable consumption. These were preferred types of solutions to more direct measures such as introducing caps or rationing systems for certain goods and services.

### Guaranteeing Minimum Levels of Needs Satisfaction

Mostly as results of the discussions of positive needs satisfiers, our forum participants proposed a range of policy ideas that can be categorized as measures guaranteeing a minimum level of needs satisfaction. In most of the forums, some type of UBI scheme was proposed, which was thought to be a way of guaranteeing satisfaction of needs such as sustenance and housing. This was seen as helping to make people feel more free and independent. There were also expectations shared by many forum participants that more time and energy should be made available for the development of social relations, civil society engagement, and mobilization for social–ecological change. For example, a 1-year sabbatical for engaging in community organizations was proposed. Furthermore, a range of suggestions aimed at broadening equal and universal access to existing and new types of UBS. Specific proposals for new UBS included free public transportation and public provision of organic food and information technology infrastructure, as well as the implementation of structures to facilitate small-scale gardening via guaranteed access to a plot of land to all interested citizens.

In a similar vein, forum participants proposed alternative forms of collective ownership in order to better provide essential goods and services to the population at large, rather than relying on market mechanisms—especially, but not only, related to the housing sector. These included proposals to share the existing urban space more equally and to increase the supply of rental apartments. Other participants wanted to see an increase in salary for (currently low-income) physically strenuous work, state guarantees for housing and work as social rights, and an abolition or reduction of fees for access to sports and other leisure activities (especially for young people). We interpret these proposals as different ways of decommodifying economy and society, put forward as alternatives to the current provision system, which was seen as too often failing to provide fundamental needs satisfaction for all.

## The Popularity of Selected Ecosocial Policy Ideas

Previous research has comparatively explored the links between social-demographic factors such as class, gender, and age and attitudes toward sustainable welfare using the European Social Survey (Fritz and Koch 2019; Otto and Gugushvili 2020) and European Values Study data (Gugushvili 2021). Here, we look specifically into ecosocial policies and ask how popular the policy proposals developed throughout the citizen forums (previous section) actually are among the Swedish population. In order to answer this question, we analyzed original survey data collected by the authors during the first months of 2021. The 11 deliberative citizen forums on sustainable needs satisfaction served as the most important input for the ecosocial policies that were included in the questionnaire. Hence, even if the forum data are not in themselves representative of the Swedish population as a whole, they were nevertheless crucial for designing the survey questionnaire. We added some proposals from the scholarly literature on degrowth, sustainable welfare, and ecosocial policies, many of which had rarely or never been made the object of survey studies. For this article, just as in the above qualitative analysis of the forum data, we focus the quantitative analysis on policies that can be seen as targeting the lower and upper limits of the safe and just operating space.

Based on a probabilistic sampling of 3000 adults from the Swedish state personal address register (SPAR), data collection was launched in the end of January 2021 and was closed in the beginning of April 2021. Data collection consisted of two rounds of postal letters containing a paper-version questionnaire and a link to the web version, with two additional rounds of postal reminders. The final response rate of the survey was 32% (951 respondents). While our respondents corresponded to the Swedish population in terms of distribution in relation to gender, geographic area, and average income level, distribution in relation to age was substantially skewed, with persons aged 60–79 years being overrepresented (a 17-percentage-point difference) and persons aged 18–39 years being underrepresented (a 14-percentage-point difference). We therefore decided to use weighted data for our analysis. Following the method developed by Lundström and Särndal (2002), the completed survey database was calibrated so that the distribution of the respondents resembled the Swedish population in terms of age, geographic area, and education. The weighted data were used in the following analyses.

The survey questions used as a basis for the following analysis are presented in Table 3. The three policy items on the upper half of the table can be seen as operationalizations of policies regulating maximum levels of needs satisfaction; in other words, these are directed toward respecting the upper boundaries of the safe and just operating space. The three items in the lower half of the table can be regarded as operationalizations of guaranteeing minimum levels of needs satisfaction or the sufficiency level, the lower boundary of this space.

**Table 3:** Survey items operationalizing “ceiling and floor” for needs satisfaction

	Survey item
Regulating maximum level of needs satisfaction (“ceiling,” “upper threshold”)	Limiting the living space per person
	Limiting the number of airline flights per person per year
	Introducing a maximum income
Guaranteeing minimum level of needs satisfaction (“floor,” “lower threshold”)	Regular distribution of a food basket with ecological and Swedish-produced raw ingredients (example for UBS)
	Free public transportation within regions (example for UBS)
	Basic income for all without obligation (example for UBI)

*Note:* Survey respondents could indicate to what extent they considered the proposals listed in the table as good or bad, using a Likert scale on which 1 = very bad and 5 = very good

*UBI* universal basic income, *UBS* universal basic services

**Fig. 1:**
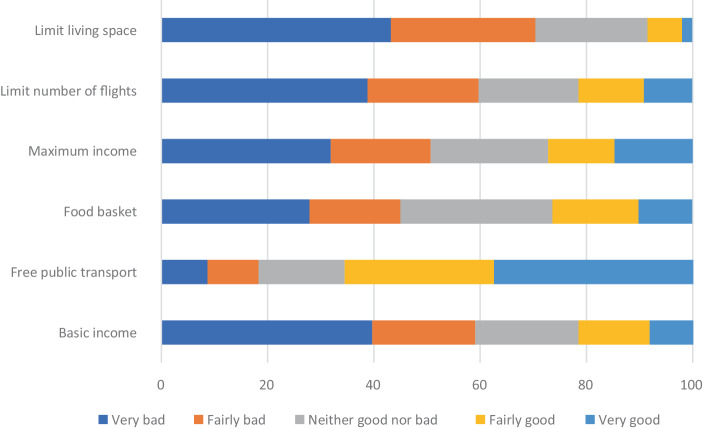
Support for policy proposals setting upper and lower thresholds (%)

Figure 1 shows that providing free public transportation within regions for all is the only proposal that the majority of our respondents considered to be positive (65.6%). Generally, the results indicate that setting upper boundaries for needs satisfaction and consumption is currently a rather unpopular policy option, since between 50% and 70% of all respondents considered these policy suggestions to be negative (70.4% against limiting living space per person; 59.7% against limiting flights; 50.7% against maximum income). This result is in line with the citizen forums, in which the participants turned out to be reluctant to discuss policy options directly limiting or constraining consumption or income level (see previous section). There is room for interpretation here, however, as to what extent the high shares of negative preferences are actually about our respondents being principally against the policy tool of “banning” something or whether they are (also) against the very outcome that the proposals are aiming for, that is, reducing the number of flights, limiting income and living space, etc. Neither of the policy proposals about guaranteeing minimum level of needs satisfaction in the areas of healthy food and income was particularly popular (26.3% for distributing a free and healthy food basket for all; 21.6% for basic income).

For all items, there are sizeable shares of respondents who considered the proposals neither positively nor negatively (19% to nearly 30% in the case of a food basket). Again, this gives cause for interpretation. One possible explanation is that the respondents choosing the neutral alternative preferred to abstain from taking a stance due to a lack of more detailed information as to how these suggestions would be applied (for instance, how they would be funded, or which regulatory frameworks might be used or created) and with what outcomes. Another possibility is that some of these proposals are simply too novel or radical for the respondents to have taken any clear-cut stance. The idea of a maximum income, for example, is not promoted by any political party in Sweden. If this situation changes, it is conceivable that support and possibly rejection rates would increase (Koch 2022a). However, another possibility is to interpret the neutral preference as a sign of widespread political indifference.

**Fig. 2:**
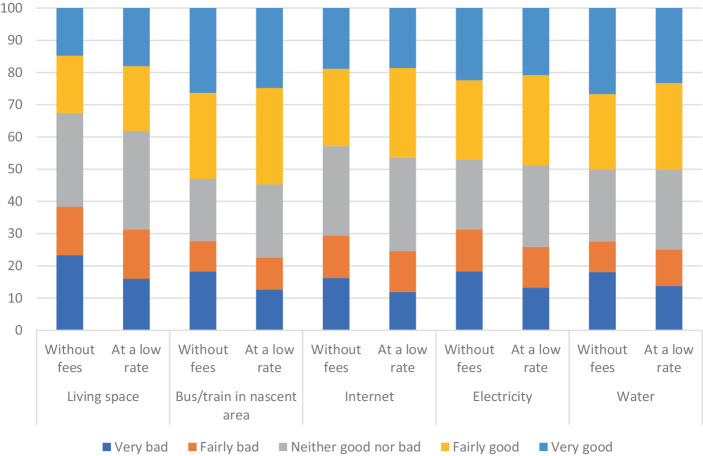
Support for basic services in five areas (%) (two alternatives: without fees and at a low rate)

As presented earlier, the provision of UBS as a way of guaranteeing a minimum level of needs satisfaction for all is gaining academic attention. This was also a theme frequently discussed in our citizen forums about sustainable needs satisfaction. In our survey, we formulated proposals entailing universal provision of essentials for five areas: X liters of water per person per year, X kilowatt hours of electricity per person per year, X gigabytes of internet per person per year, X kilometers of public transportation per person per year, and X square meters of living space per person per year. Figure 2 depicts what our respondents thought about guaranteeing basic levels of essential services to all at no or a very low cost.

Large shares of our respondents considered the provision of guaranteed levels of essential services and goods as something positive (“very good” or “fairly good”): around 50% for water, 47%–48% for electricity, 44%–46% for internet, and 53%–55% for public transportation (figures differ slightly between the options “with no fees” and “at a low rate”). What sticks out here is that, compared to the other areas, the idea of guaranteeing a minimum amount of living space for all seems to be relatively unpopular. This is also the policy suggestion where we find the largest difference among the general supporters of a proposal, in this case between the two options “with no fees” and “at a low rate”: 32.6% and 38.2%, respectively. In relation to all other proposals, this difference is around 2%. One further noticeable aspect is the high share of respondents who found the proposals neither good nor bad, ranging from 20% to nearly 30% (the grey part of each bar). This rather high percentage of the “undecided” option resembles answers to the six questions on upper and lower limits (Fig. 1). In our citizen forums, participants had the opportunity to discuss possible motives for such basic provision of services, not only from the perspective of universal welfare provision but also as a way of contributing to an enabling societal condition for wider political participation and civic engagement. In the survey format, however, there is no such room for elaboration, and this may be attributed to the high share of neutral preferences expressed by the respondents. On the other hand, this high share of neutral policy preferences could be regarded as a social basis for potential policy support.

## Discussion and Conclusion

Given the impossibility of ecologically generalizing the Western welfare–work nexus as it developed in the post–World War II context to the rest of the world, we set out to theoretically and empirically explore the potentials for a democratically deliberated, sustainable welfare–work nexus in a postgrowth context in Sweden. Theoretically, we built on the concepts of sustainable welfare and a safe and just operational space within which needs satisfaction may be established within planetary and social limits. Hence, the emerging “sustainable welfare–work nexus” would be at the core of moving production and consumption patterns toward the space between lower or sufficiency levels of basic needs satisfaction and upper or planetary limits of needs and wants. The empirical work was oriented at deliberating ways of understanding, operationalizing, and politically targeting both upper and lower boundaries of that space and exploring the popularity of corresponding “ecosocial” policies.

The qualitative forum material revealed rather widespread dissatisfaction with the ways existing policy frameworks address the ecological and social crises in Sweden. It also points to a certain willingness to address these crises head on and from the bottom up. The human needs perspective turned out to be a suitable lens through which sustainability issues may be deliberated and policy ideas could be developed. Although the needs matrix by Max-Neef initially came across as somewhat abstract, most participants went on to utilize it effectively in discussing a range of social, economic, and environmental issues and policy ideas in connection to sustainable needs satisfaction. The needs matrix supported the identification of negative and positive needs satisfiers as well as so-called bridging and synergetic needs satisfiers, which can be translated into policies capable of moving economy and society toward respecting the upper and lower boundaries of the safe and just operating space. In relation to the lower boundary, both UBI and, especially, UBS schemes were broadly endorsed, while, in relation to the upper boundary, the notion that not all wants can be supported in an ecologically constrained world met general approval. A range of corresponding policy proposals were suggested and discussed.

The results of the quantitative survey, which was to a large extent designed on the basis of the forum policy suggestions, however, point to a considerable gap between the far-reaching policy measures that most forum participants as well as the scientific literature consider necessary to meaningfully address the environmental crisis (see introduction) and the measures that citizens of an advanced welfare state such as Sweden are presently prepared to support. Approval rates for guaranteeing minimum needs satisfaction levels or respecting the lower boundary of the safe and just operating space—and here for UBS schemes in particular—turned out to be much higher than those for policy ideas to regulate maximum satisfaction levels of needs and wants. Any understanding of why UBS solutions are so much more popular in Sweden than UBI would need to consider the rather articulated work ethic, according to which full-time employment is expected for men and women, as well as consider the traditional strong universal tradition of welfare delivery in the Nordic countries, as a result of which people may find it easiest to expand these existing systems. However, in countries where UBS exists in merely rudimentary forms and liberal welfare traditions predominate, UBI may be the easiest and quickest option for proceeding.

Explanations for the hesitation to implement policies targeting maximum levels of needs satisfaction, luxuries, or the “upper boundary” or the safe operating space include the thorough inculcation of the existing economic and social order, including the growth imperative in people’s minds, bodies, and day-to-day social practices (Koch 2018) often appearing as natural differences and the only possible way of doing things. Indeed, a significant share of the electorate seem to believe in the “trickle-down” effect: to not regulate economic growth, and the rich will be in the interest of the poor as well (Fritz et al. 2021). In addition, since it is part of the collective consciousness that a range of institutions—such as the legal, educational, and welfare systems, which have proven to be crucial for the relatively high subjective well-being scores measured in Western societies—historically codeveloped with the provision of economic growth and are presently coupled to it, any political move beyond the capitalist growth economy would need to reckon with concerns about well-being loss, anomie, and social exclusion (Büchs and Koch 2017).

One way to defuse these concerns could be to expand already existing spaces, where alternative, sustainable, and cooperative forms of working and living together are tested. Our citizen forums are an example of such alternative spaces. However, for these to become effective in relation to social–ecological change, it would be necessary to carry out participatory exercises (such as workshops, consultations, or deliberative forums) much more often and on greater scales than in our case (Guillén-Royo 2020).^3^ Beyond deriving critical input for research and legitimacy for policymaking, such deliberative and participatory exercises could function as an educational opportunity for the wider citizenry. For instance, they could help to introduce and make popular concepts such as “planetary boundaries” and the “sufficiency principle,” which are capable of challenging the policy paradigms in our current growth-oriented economic system. Presently, we can only speculate whether public support for the suggested ecosocial policies in our survey study would increase if a significantly greater share of the Swedish population took part in similar deliberative forums and had a chance to reflect on the current ways of satisfying our fundamental needs within planetary limits. Yet our results appear promising enough to recommend that policymakers in established democracies launch corresponding deliberative attempts.

As recent Irish and French experiences with “climate assemblies” indicate (Harris 2021), governments could support participatory exercises by enhancing the status of citizen forums and giving them an advisory character. This would be in line with recent political science reasoning that an adequate response to the ecological crisis requires augmenting the institutions of representative democracy with mechanisms of direct and deliberate democracy that carry the potential of “disruptive deliberation” (Hammond 2020), a theme taken up by other papers in this special issue. Future research could further explore the preconditions under which and the extent to which deliberative practices and reforms may expand the social base for social–ecological transformations and to democratically move economy and society toward a safe and just operating space. Policymakers may support this by tackling the inequality and environmental crises at the same time and by showing willingness to act upon corresponding bottom-up policy suggestions.

## Footnotes

1 The concept of sustainable “consumption corridors” (Fuchs et al. 2021) has a similar intention and focus.

2 For methodological details of HSD and further workshop results, see Lindellee et al. (2021) and Koch et al. (2021).

3 Hardly anyone in the forums articulated fundamental doubts about the Swedish democracy as such. However, many emphasized a need for a complementation of the existing representative democracy by elements of a more direct kind, including citizen forums. This applies to the generation of concrete policy ideas and the more general involvement of citizens in the social–ecological transformation process.
